# Retrieving similar substructures on 3D neuron reconstructions

**DOI:** 10.1186/s40708-020-00117-x

**Published:** 2020-11-04

**Authors:** Jian Yang, Yishan He, Xuefeng Liu

**Affiliations:** 1 Faculty of Information Technology, Beijing University of Technology, Beijing, China; 2 Beijing International Collaboration Base On Brain Informatics and Wisdom Services, Beijing, China; 3 School of Artificial Intelligence, University of Chinese Academy of Sciences, Beijing, China

**Keywords:** Neuronal morphology, Reconstruction, Substructure, Retrieving

## Abstract

Since manual tracing is time consuming and the performance of automatic tracing is unstable, it is still a challenging task to generate accurate neuron reconstruction efficiently and effectively. One strategy is generating a reconstruction automatically and then amending its inaccurate parts manually. Aiming at finding inaccurate substructures efficiently, we propose a pipeline to retrieve similar substructures on one or more neuron reconstructions, which are very similar to a marked problematic substructure. The pipeline consists of four steps: getting a marked substructure, constructing a query substructure, generating candidate substructures and retrieving most similar substructures. The retrieval procedure was tested on 163 gold standard reconstructions provided by the BigNeuron project and a reconstruction of a mouse’s large neuron. Experimental results showed that the implementation of the proposed methods is very efficient and all retrieved substructures are very similar to the marked one in numbers of nodes and branches, and degree of curvature.

## Introduction

The brain is made up of a complex network of billions of neurons, and is one of the most important and complex organs in the body. To investigate the neural mechanism of brain functions and explore the pathogenesis of brain disorders, some country-level large projects were launched, such as US BRAIN Project, European Human Brain Project (HBP), Japan Brain/MIND Project and China Brain Project [1]. Neuronal morphology plays a prominent role in the investigation of neuronal structure and function, which is determined by a number of factors, including physical and biological constraints and requirements of axonal, dendritic, and so on [2]. One of the fundamental tasks or preliminary work of above brain projects is seamlessly reconstructing and aggregating neuronal morphologies on scales up to the whole rodent brain. Many computer-based computational methods and tools have been developed for tracing a single neuron from 3D digital microscopy image stacks [3–7]. Many existing tracing methods can generate overall good reconstructions, but perform poorly on some local substructures because of noises in digital images or the complexity of neuronal morphologies. One way to improve the accuracy of reconstructions is marking some typical inaccurate substructures, retrieving and checking their similar substructures one by one. In addition, some morphological substructures of a neuron are highly correlated to its function or category, which should be receive high interest. In this work, we propose a pipeline to retrieve most similar substructures to a marked substructure on one or more neuronal reconstructions.

Recent advances in microscopic imaging systems have made it possible to collect large-scale digital images of neurons. Reconstructing neuronal morphology can help biologists to visualize and study cellular structures [7]. Automatic tracing methods have been investigated for more than 20 years, and neuron reconstruction has become a hot topic in computational neuroscience [8]. Two projects greatly promoted its development: the DIADEM (short for digital reconstruction of axonal and dendritic morphology) neuron reconstruction challenge held in 2010 [9, 10] and the BigNeuron project launched in 2015 [11]. Many automatic tracing methods based on different principles and models have been proposed, such as automatic contour extraction [12], APP1 [13], Open-Curve Snake [14], Ray casting [15], APP2 [16], MOST [17], tTuFF [18], Rivulet [19], SparseTracer [20], Ensemble neuron tracer [21], and so on. Automatic tracing methods are usually divided into categories: global processing and local processing approaches, and Acciai et al. labeled them as three categories with additional meta-algorithm approaches [7]. Global approaches process whole images, the local ones explore an image only around relevant structures, and meta-algorithm approaches enhance existing methods in some aspects to manage large-scale images [7].

**Fig. 1:**
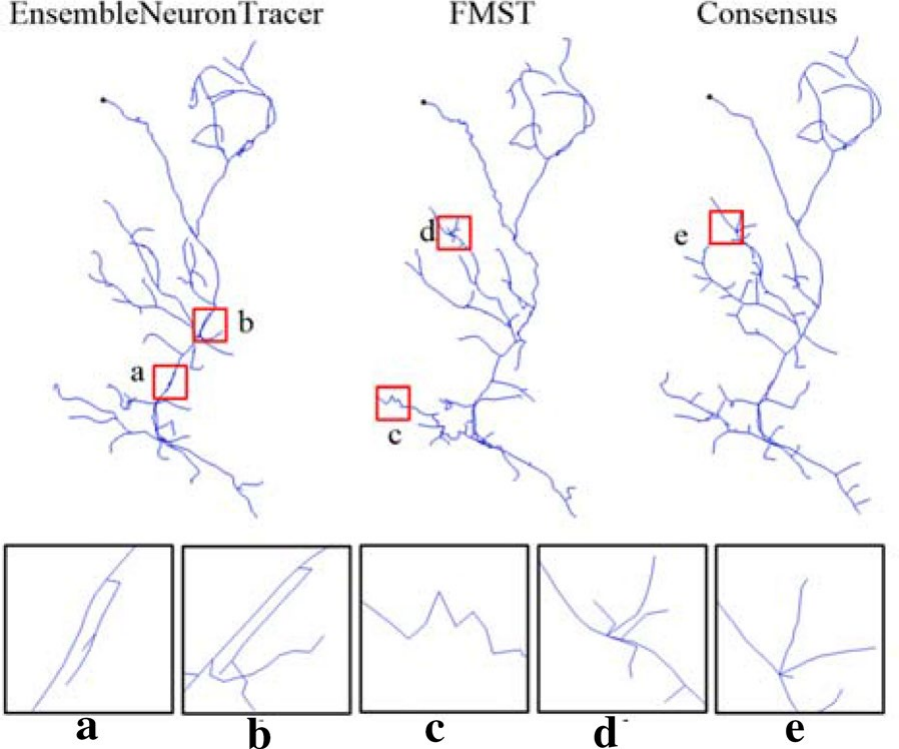
Inaccurate substructures on three reconstructions of a neuron. Reconstructions generated by Ensemble Neuron Tracer, FMST and a consensus strategy have some inaccurate substructures marked by **a**–**e**, which have too many branches or zigzags

Automatic tracing methods developed for different application scenarios, and based on different models and strategies typically have varying performance [11]. An automatic tracing method may perform well on most of a neuron, but fail to capture local substructures in some small regions. For example, in Fig. 1, three reconstructions generated by Ensemble Neuron Tracer [21], FMST [22] and a consensus strategy have some inaccurate substructures with too many branches or zigzags. To produce a reconstruction with high accuracy, we may generate a reconstruction using an automatic tracing method, and then check and revise it manually. If an inaccurate substructure is found, all its similar substructures need to be retrieved from the reconstruction and mended one by one. In addition, some existing studies showed that certain morphological substructures of a neuron are important for investigating its function or categorization. Dendritic morphology helps to define the size and interdependence of functional compartments in a neuron [23]. The uncoupling of soma from the dendrites in Purkinje cells, or from the apical dendrites in thick-tufted pyramidal cells, significantly impacts various features of somatic firing and synaptic integration [24–26]. Morphologies of neurons in a category may vary in overall shape and size, but they probably have some very similar (common) substructures which are called neuron morphology motifs [27]. Retrieving these motifs from all neurons in the category is helpful for characterizing their common features and investigating their common functions.

Query-based retrieval of relevant neurons from a database has been studied recently, which is important for comparative morphological analysis, neuron classification and relationship investigation between neuronal structure and function [28, 29]. Costa et al. employed pairwise 3D structural alignments to search similar neurons [30]. Polavaram et al. evaluated morphological similarities and dissimilarities between groups of neurons by deploying unsupervised clustering technique and using expert-labeled meta-data (like species, brain region, cell type, and archive) [31]. Wan et al. designed BlastNeuron as a software pipeline for automatic retrieval and comparison of neuron morphology in a 3D neuron reconstruction database. BlastNeuron retrieves similar neurons for a query neuron in two steps: calculating the similarity between the query and candidate neurons using global morphological features, and finding their local spatial alignments [27]. Conjeti et al. presented a tool called Neuron-Miner, for fast reference-based retrieval within neuron image databases [29]. The kernel algorithm in Neuron-Miner is hashing forests, which is based on the hashing (searching and retrieving) technique and employs multiple unsupervised random trees. As far as we know, there is no study on retrieving morphological substructures on a neuron reconstruction. Though substructure retrieval and neuron retrieval may share some ideas or strategies, they have two differences: different representation of the query structure and different searching space, which lead to the need for exploring substructure retrieval independently. For neuron retrieval, the query structure is a neuron tree used as the reference and its searching space is all neurons in the used dataset. For substructure retrieval, substructure may be provided via several points on its boundary and is not an explicit tree structure, and its search space is all nodes of one (retrieving on only one neuron) or more neurons (retrieving on a neuron dataset).

If we are interested in a substructure on a neuron reconstruction, a region around it in the space of the neuron reconstruction is marked and stored via several points on its boundary. The marked region is always anisotropic (neither a sphere nor cube), which makes it difficult to construct a query substructure and construct candidate substructures. The neuronal structure contained in the axis-aligned minimal bounding box (AABB) of the marked region is taken as a marked substructure, and the marked structure is extended using its topological property. We propose a pipeline to retrieve most similar substructures for a query substructure, and implement it as a plugin of Vaa3D [32, 33]. Experimental results on 163 gold standard reconstructions provided by the BigNeuron project and the reconstruction of a mouse’s large neuron showed that the proposed pipeline can efficiently and effectively retrieve substructures most similar to the query.

## Method

For a single tree reconstruction, we design a method, called maximum subtree (MS) to construct a query substructure and implement the retrieval procedure. The MS method takes the maximum subtree with same center and radius to the marked substructure as a query substructure, and then constructs a candidate substructure at each node on the tree, which is the maximum subtree centered at the node with the query’s radius.

### Overview of retrieving similar substructures

**Fig. 2:**
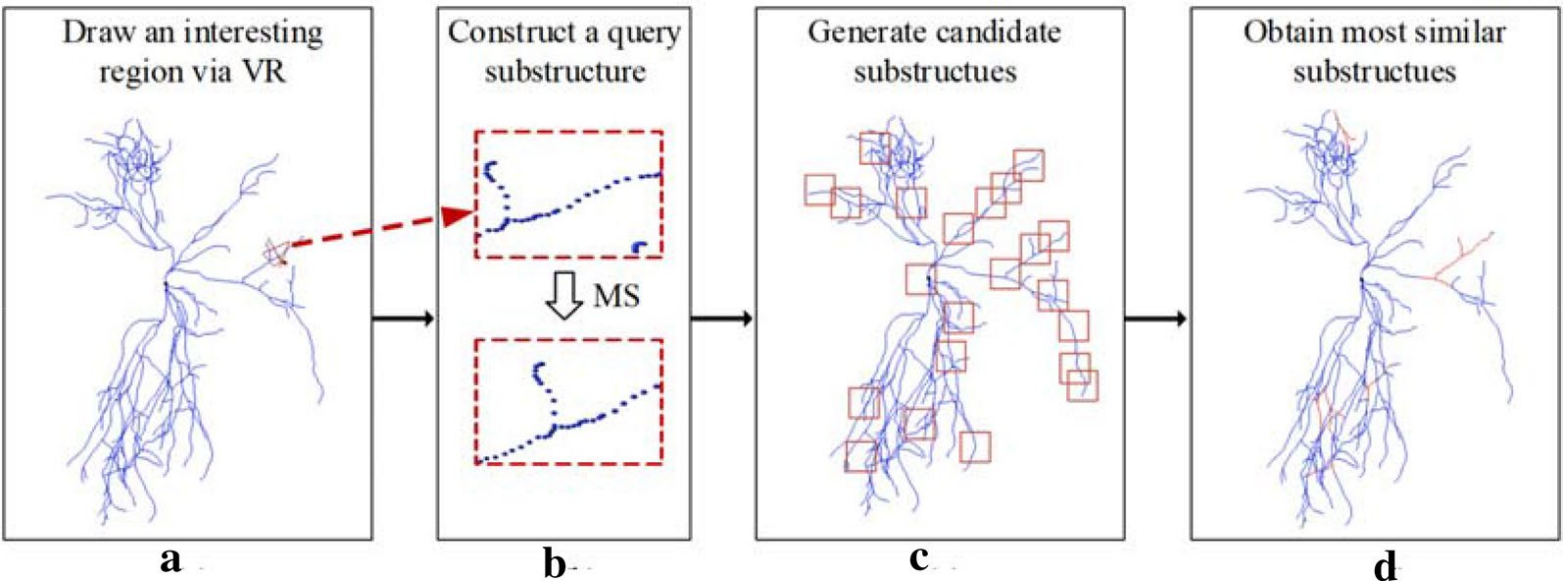
The workflow of the proposed pipeline. **a** A reconstruction of a neuron and an interesting region (in red) on it which was drawn via VR plugged in Vaa3D. **b** The marked substructure in the interesting region (top), and its query substructures constructed by MS (bottom). **c** Some candidate substructures boxed on the reconstruction. **d** Some retrieved substructures (in red) demonstrated on the reconstruction, which are most similar to the query

The workflow of the proposed pipeline is demonstrated in Fig. 2. It consists of four steps: getting a marked substructure (Fig. 2a), constructing a query substructure (Fig. 2b), generating candidate substructures (Fig. 2c), and retrieving most similar substructures (Fig. 2d). For a neuron reconstruction, we manually draw the boundary of an interesting region in the neuronal 3D space via a virtual reality device (VR), which is implemented as a plugin of the Vaa3D platform [34]. The boundary of the marked region is described by three-dimensional coordinates of several points on it and saved as a SWC file [35]. The neuronal structure contained in the AABB of these points is taken as a marked substructure, and a query substructure and its candidate substructures at each candidate node are constructed by the MS method. Then the similarity between the query and candidate substructures is calculated using 19 quantitative morphological features, and substructures most similar to the query are taken as retrieved substructures. Retrieved substructures can be demonstrated on the neuron tree, and checked and revised one by one manually.

### Maximum subtree (MS) method

The marked substructure is extracted from the AABB of the marked region and may consist of more than one unconnected subtrees. MS only considers the subtree with most nodes (denoted by $S$) and uses $S$ to construct a query substructure. Two main steps of MS are calculating the center and radius of $S$, and generating a maximum subtree with the radius as the query or a candidate substructure.

The definitions of radius and center of a graph and a related theorem are introduced as the theoretical base for the calculating step. Let $G=\left(V,E\right)$ be a connected graph with node set $V$ and edge set $E$, then the eccentricity $\varepsilon (v)$ of a node $v$ is the maximum shortest distance between $v$ and any other node in $V.$ The radius $\mathrm{rad}\left(G\right)$ and diameter $\mathrm{diam}\left(G\right)$ of *G* are defined as the minimum and maximum node eccentricity of $G$, respectively. The center $C(G)$ of $G$ is the set of nodes with eccentricity equal to $\mathrm{rad}\left(G\right)$. If $G$ is a tree, $C(G)$ contains at most two nodes and has the following property [36].

#### Property 1

[36]. Let $L$ be the set of leaves of a tree $G=(V,E)$. If $|V|\le 2,$ $L$ is the center of $G$, otherwise the center of $G$ remains the same after removing of $L$: $C\left(G\right)=C(G\backslash L)$.

This property brings us to an algorithm for finding the center of a tree: removing leaves of a tree level by level until no more than 2 nodes remain. After running the algorithm by the breadth first search (BFS) strategy, remaining nodes are components of the center and we have

1 $$\mathrm{diam}\left(G\right)=2*\mathrm{maxlevel}+\left|C\right|-1\mathrm{ and rad}\left(G\right)=(\mathrm{diam}\left(G\right)+1)/2,$$

where maxlevel is the maximum number of levels executed in the algorithm and $\left|C\right|$ is the number of nodes in $C\left(G\right)$. So we can get the center $C\left(S\right)$ and radius $\mathrm{rad}\left(S\right)$ of $S$ by implement the above algorithm and formula (1), respectively.

In the generating step, we also use the BFS strategy to construct the maximum subtree centered at a node $x$ with radius $\mathrm{rad}\left(S\right)$, where $x$ is a node in $C\left(S\right)$ (for the query substructure) or any candidate node on the whole neuron tree (for a candidate substructure). We set two sets $I$ and $B$, and initialize $I$ as empty and $B$ as $\{x\}$. For each node $y$ in $B$, if its distance to $x$ is smaller than $\mathrm{rad}\left(S\right)$, it is transferred from $B$ to $I$ and all its child nodes and parent node are put into $B$; if its distance to $x$ is equal to $\mathrm{rad}\left(S\right)$, it is transferred from $B$ to $I$. This process is repeated until $B$ is empty, and then $I$ contains all nodes of the maximum subtree centered at $x$ with radius $\mathrm{rad}\left(S\right)$. MS takes the maximum subtree as the query substructure or a candidate substructure at a candidate node, respectively.

### Retrieving most similar substructures

After a query substructure and some candidate substructures on one or multiple reconstructions are obtained, we compare their morphological features and pick out candidate substructures most similar to the query. In BlastNeuron [27], Wan et al. designed a “global search” method to search morphologically similar neurons in a large database of neuron reconstructions, which compares 3D neuron reconstructions using global morphological features and moment invariants. The “global search” method performed well on the entire database of NeuroMorpho.org, so we utilize it to retrieve most similar substructures in our pipeline. Substructures always have simple small tree structure and possibly more than one subtrees, which is different from a whole neuron reconstruction. We use 19 global morphological features (except average local amplitude angle and average remote amplitude angle in BlastNeuron, which may not make sense for substructures without bifurcation) to calculate the similarity between a query and a candidate substructure. These 19 morphological features (Table 1) are selected from the function list of the L-measure software (https://cng.gmu.edu:8080/Lm/help/index.htm) and are invariant to translation and rotation of the neuron [27, 37].

**Table 1:** List of global morphological features

Number of nodes	Soma surface area
Number of stems (branches on cell body)	Number of bifurcations
Number of branches	Number of tips
Neuronal height	Neuronal width
Neuronal depth	Total length
Total surface area	Total volume
Maximum branch order	Maximum Euclidean distance to root
Maximum path distance to root	Average contraction
Average diameter (thickness)	Average fragmentation
Average parent–daughter ratio	

## Experimental results

In our experiment, we tested the proposed pipeline on a small neuron, a large neuron and a morphology database. An interesting region on a neuron can be marked by a VR device plugged in the Vaa3D platform. Since our experiments need many interesting regions on many neurons, we randomly selected them by giving some points on their boundaries. The morphology database is gold166 bench-testing neuron reconstructions (https://github.com/BigNeuron,163 neurons expecting 3 neurons without gold standard reconstruction), which contains reconstructions of 8 chick neurons, 2 frog neurons, 91 fruit fly neurons, 11 human neurons, 31 mouse neurons, 7 silkmoth neurons and 13 zebrafish neurons. For each neuron, there are one gold standard reconstructions traced by human experts and 40 + reconstructions generated by 20 + automatic tracing algorithms. The small neuron was selected from the gold166 dataset and the large neuron is a mouse neuron with 80,000 + nodes.

### Retrieving inaccurate substructures in an automatic tracing reconstruction

**Fig. 3:**
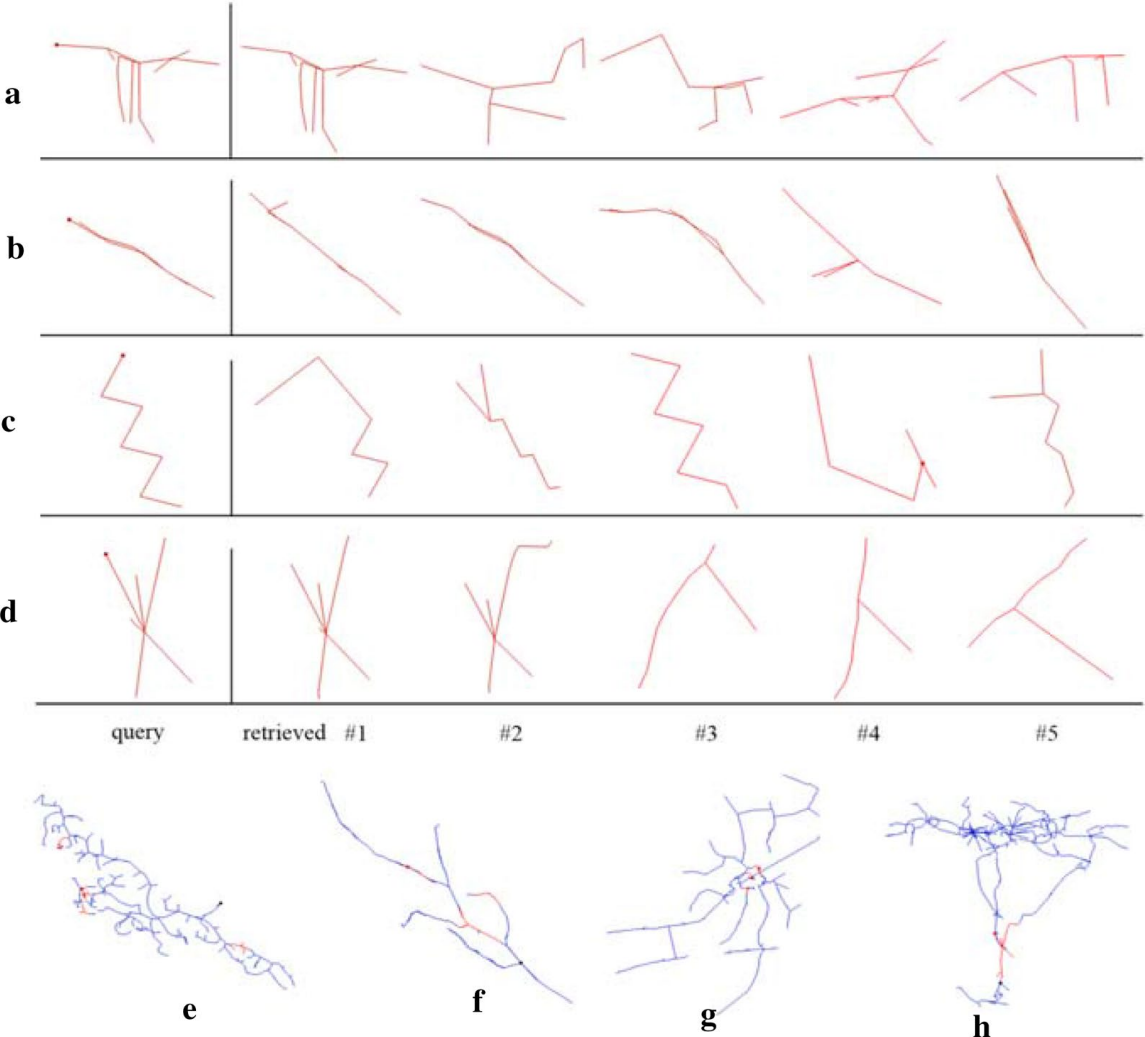
Retrieved inaccurate substructures on a reconstruction. **a**–**d** are the query and its five retrieved substructures on reconstructions **e**–**h**, respectively

We first selected some automatic tracing reconstructions from the gold166 dataset, and marked some inaccurate substructures on them by visual check, which have too many branches, zigzags, or two long parallel branches. Then a query was constructed based on each selected inaccurate substructure, and its most similar substructures in the reconstruction were retrieved. The query and five retrieved substructures on each of four reconstructions are illustrated in Fig. 3. Subfigures (a)–(d) are the query and its five retrieved substructures on reconstructions (e)–(h), respectively. Reconstructions (e)–(h) are: a fruit fly neuron traced by APP1, a human neuron traced by NeuronChaser, a mouse neuron traced by NeuronStalker, and a zebrafish neuron traced by MOST. It can be seen that those retrieved substructures are quite similar to the corresponding query, and they are also inaccurate substructures and need to be checked and amended manually.

### Retrieving similar substructures in a single neuron

To avoid falling into the discussion of what is inaccurate or the important substructure in a neuron, we randomly selected a number of substructures to demonstrate the validity of the proposed pipeline in retrieving similar substructures. The pipeline was implemented on all 163 neurons in gold166 dataset and one large mouse neuron.

**Fig. 4:**
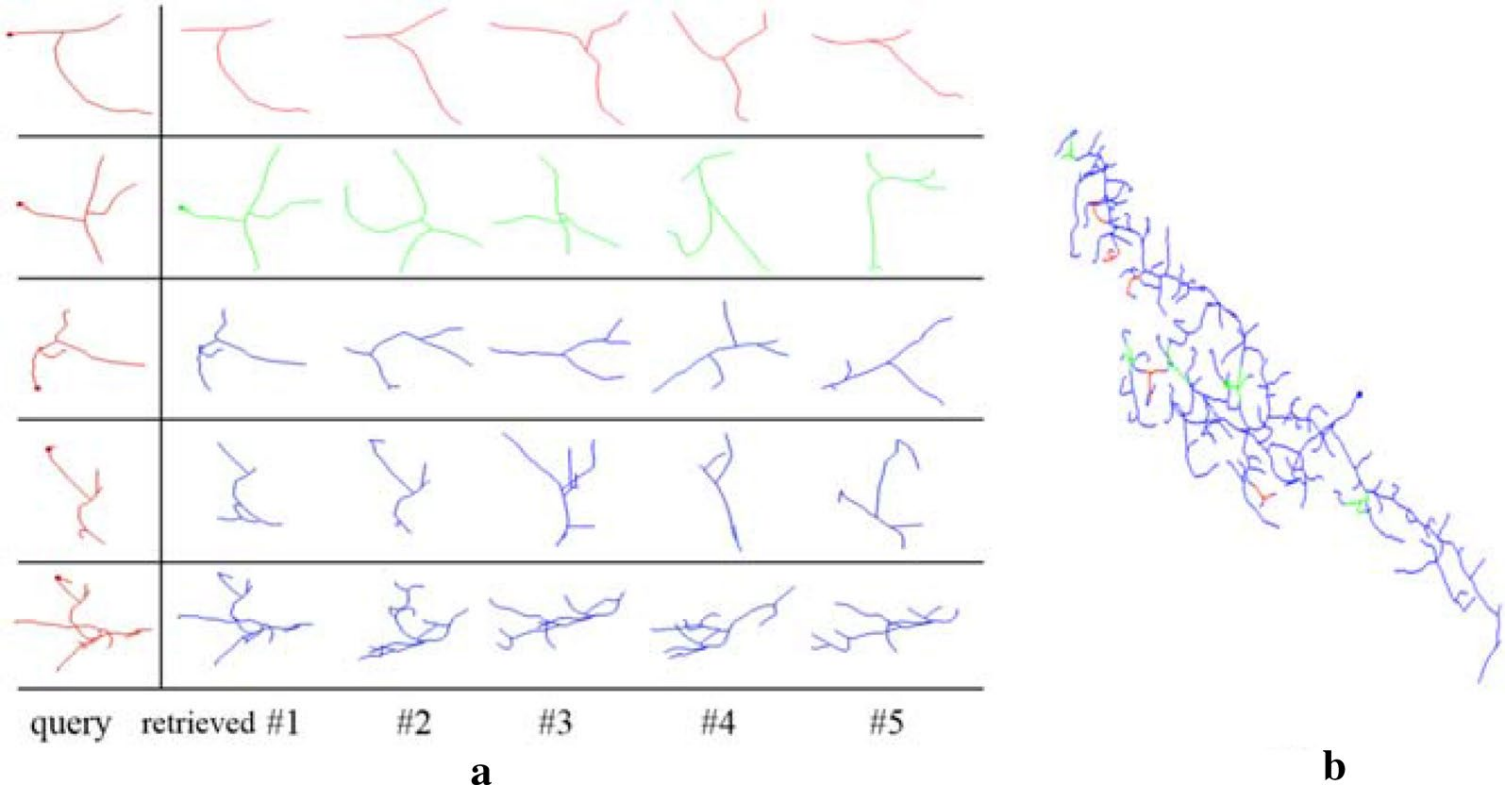
Retrieved substructures on a fruit fly neuron. **a** Each row gives a query substructure and its five most similar substructures on the neuron retrieved by MS. **b** Substructures in the first two rows of **a** are demonstrated on the neuron (substructures of the first row are in red and the second are in green)

Five regions in each of these 163 gold standard reconstructions were randomly marked to construct queries and their most similar substructures in the neuron were retrieved by the proposed pipeline. Five queries on a fruit fly neuron are given in the left column of Fig. 4a, and their retrieved five most similar substructures were given in five columns on the right. Substructures in the first two rows of Fig. 4a are demonstrated on the neuron in Fig. 4b. It can be seen that retrieved substructures are similar to the query in numbers of nodes and branches, and degree of curvature. So MS is capable of extracting the structure information of the query and using it to construct candidate substructures on a small neuron tree.

**Fig. 5:**
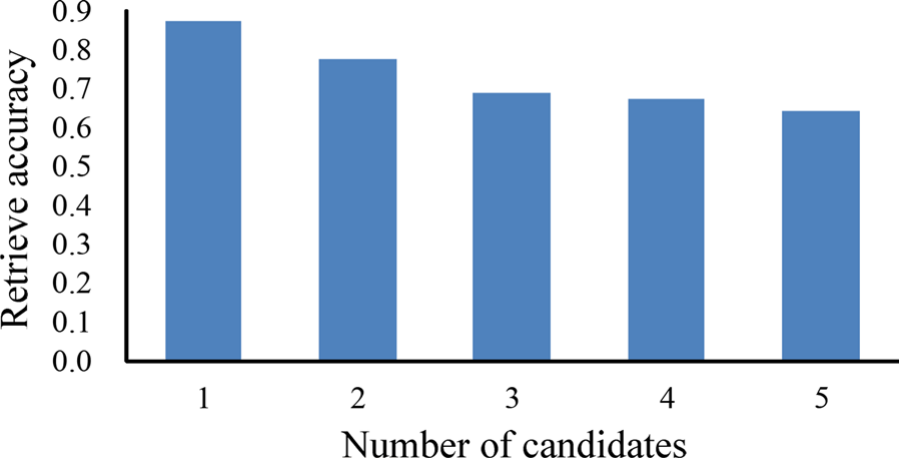
Accuracy of the first one to five retrieved substructures compared to the query

Each query substructure together with its 5 retrieval results were visually inspected and compared on Vaa3D platform. For most of 163*5 = 815 query substructures, our pipeline successfully retrieved 3–4 morphologically similar substructures (Fig. 4). Visual comparison done by three independent people show that 84% (685/815) of top 1 retrieved substructures are really similar to the corresponding query substructure. Furthermore, the accuracies corresponding to top 1 to 5 retrieved substructures are plotted in Fig. 5. The accuracy decreases as more candidates were included, yet it is always above 60% if five retrieved results are considered. This indicates that our method is capable of finding similar substructures on a neuron effectively.

**Fig. 6:**
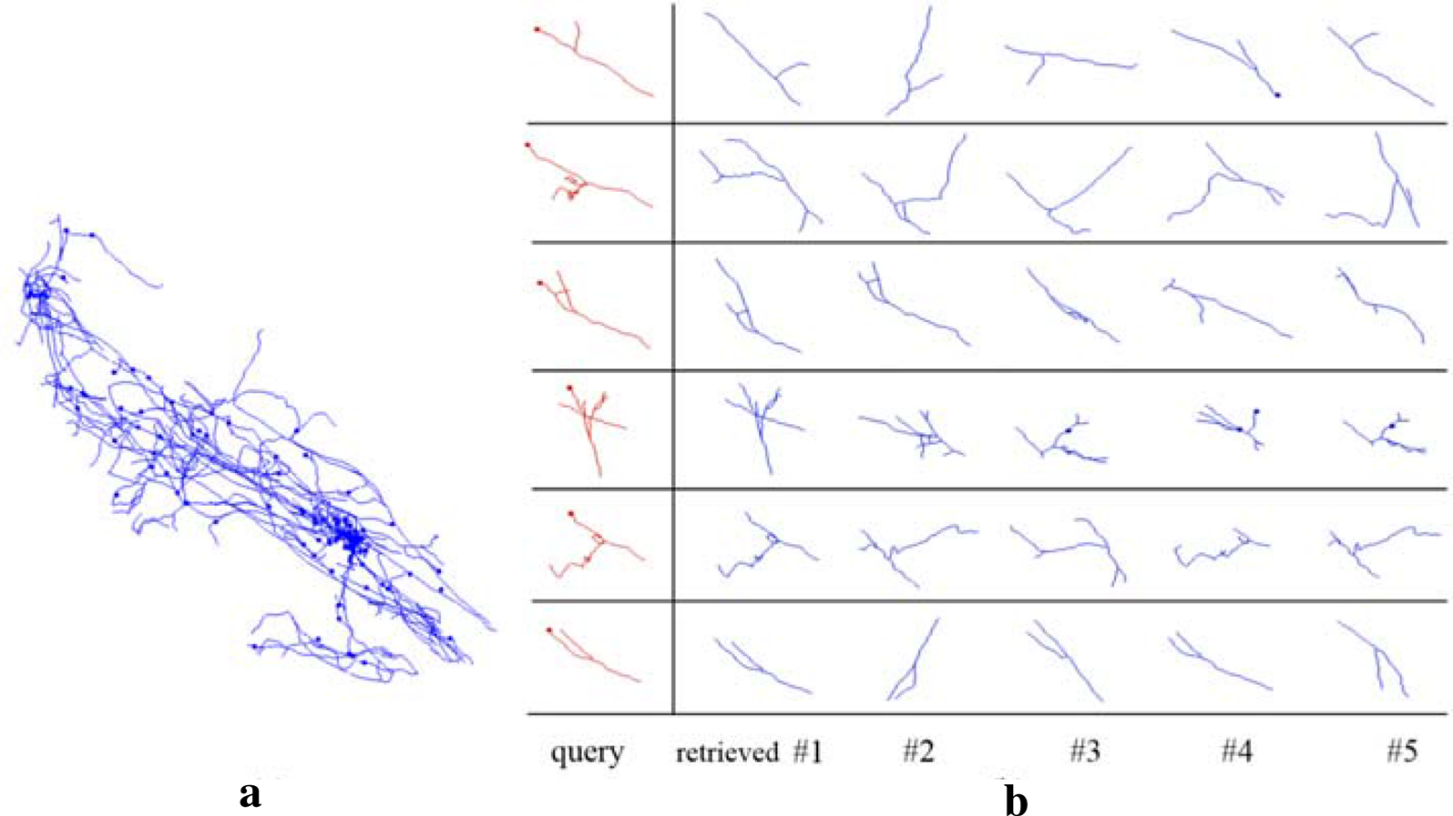
Query substructures (left column) on a large neuron (80,000 + nodes) and their top 5 similar substructures retrieved by MS

The reconstruction demonstrated in Fig. 6a is a reconstruction of a large mouse neuron provided by the Southeast University-Allen Institute Joint Center, which was manually drawn in Vaa3D by a human expert. The reconstruction has more than 80,000 nodes and consists of more than 1000 unconnected segments, which means there are more than 1000 root nodes. To implement MS, a preprocessing was implemented on the reconstruction to connect and sort it to one tree. Six query substructures (in red) and their top five similar substructures (in blue) retrieved by MS are demonstrated in Fig. 6b. We can see that retrieved substructures are very similar to their corresponding queries. The pipeline can effectively retrieve similar structures on the preprocessed reconstruction. The implementation of MS was quite efficient, and its running time on our laptop was 1 min for one query on this large neuron.

### Retrieving substructures in a morphology database

**Fig. 7:**
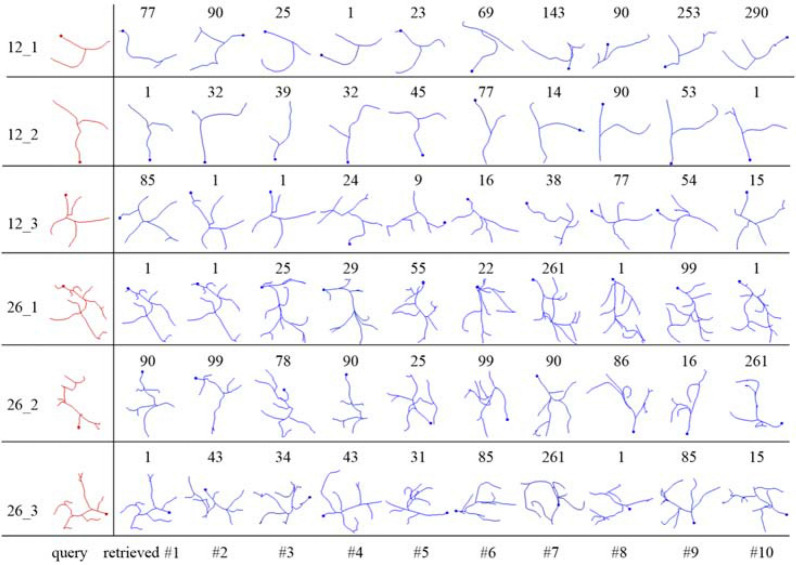
Retrieved substructures on gold standard reconstructions in gold166 dataset. Three queries from two neurons (numbered 12 and 26) are in red, and their top 10 similar substructures retrieved by MS are in blue. The number above each substructure is the number of the neuron in which the retrieved substructure locates

To investigate a key substructure in a set of neurons, we need to know in which neuron or where it locates. That is to say, with a given query, its most similar substructures are needed to be retrieved from all neurons in the set. The proposed procedure was used to retrieve a query’s most similar substructures in all 163 gold standard reconstructions. Three queries (in red) from two neurons (neuron numbered 12 and 26) and their top 10 retrieved results by MS (in blue) are given in Fig. 7. It can be seen that most similar substructures were successfully found from different neurons. Multiple retrieved substructures may come from one neuron, and many neurons might have no retrieved substructures. The number of retrieved substructures a neuron has depends on the degree of the similarity between its local morphology structure and the query.

## Conclusions and discussion

We designed a pipeline to rapidly retrieve similar substructures on neuron reconstructions, actualized it as a plugin of the Vaa3D platform, and tested them on 163 small neurons and a large neuron. Experimental results show that the proposed method can successfully and quickly retrieve most similar substructures on one or more neuron reconstructions. Retrieved substructures have similar numbers of nodes and branches, and similar degree of curvature to the query. And the top 1 retrieved substructures is usual the query itself.

Our proposed pipeline supplements the study of neuronal morphology retrieval. Neuroscientists may use Neuron-miner [32] and Blastneuron [27] to retrieve similar neurons in a neuronal morphology database for a query neuron, and utilize our method to search for similar substructures on a neuron or multiple neurons. The neuronal morphology and substructure retrieval make the use of neuron data more conveniently and can promote the study of morphology based neuronal classification and function.

Our MS method utilizes attributes of tree structure to eliminate the irregularity of a marked region, and is easy and fast to implement. It uses the concept and property of a tree’s center and radius in graph theory to construct substructures, and employs the BFS algorithm to implement the construction. It spent less than ten seconds to retrieve a substructure on a neuron reconstruction with 1000 nodes. For a large reconstruction with 80,000 + nodes, it took one minute on our laptop. However, MS is incapable of handling unconnected neuron reconstruction and searching substructures with multiple subtrees or segments.

While constructing candidate substructures on a neuron, we need to traverse all nodes on the neuron. If we have enough computational time and want to obtain all similar substructures, all nodes can be selected as candidate nodes one by one. Otherwise, we go through the SWC file of the neuron by a given step. Retrieved substructures change a little with different length of steps. If the step is bigger, less candidate substructures are constructed and some most similar candidate substructures might be missed. If the step is smaller, more candidate substructures are constructed and the calculating time is longer. For large neurons, an appropriate step is needed to balance the running time and the retrieval performance.

The 19 morphological features used to calculate the similarity between a query and candidate substructures reflect global morphological character. Since subtrees in a substructure are relatively much smaller than a whole neuron tree and a substructure may contain multiple subtrees, global features might not be optimal for characterizing the local morphology. The proposed pipeline will be further improved by extracting some new features and designing a more sophisticated similarity for substructures on a neuron.
